# Method of Characterization and Classification of the Physicochemical Quality of Polished White Rice Grains Using VIS/NIR/SWIR Techniques and Machine Learning Models for Lot Segregation and Commercialization in Storage and Processing Units

**DOI:** 10.3390/foods15010062

**Published:** 2025-12-24

**Authors:** Letícia de Oliveira Carneiro, Nairiane dos Santos Bilhalva, Ênio Antônio Manfroi Filho, Dthenifer Cordeiro Santana, Larissa Pereira Ribeiro Teodoro, Paulo Eduardo Teodoro, Paulo Carteri Coradi

**Affiliations:** 1 Laboratory of Postharvest (LAPOS), Campus Cachoeira do Sul, Federal University of Santa Maria, Avenue Avenue Taufik Germano, 3013, Universitário II, Cachoeira do Sul 96503-205, Rio Grande do Sul, Brazil; leticiaoc.eng@gmail.com (L.d.O.C.);; 2 Digital Agriculture Laboratory (LaDi), Campus de Chapadão do Sul, Federal University of Mato Grosso do Sul, Chapadão do Sul 79560-000, Mato Grosso do Sul, Brazil

**Keywords:** *Oriza sativa* L., commercial batches, reflectance spectroscopy, spectral bands, predictive models, MLP

## Abstract

The quality of rice depends on physical, nutritional, and sensory attributes. However, in industrial practice, quality is predominantly based on physical characteristics evaluated by the conventional method for categorizing commercial atches. In this context, the present study aimed to characterize the physical quality and proximate composition and to classify commercial batches of polished white rice using machine learning (ML) algorithms based on spectral data. Individual samples (healthy grains and physical defects) and samples from commercial batches (Type 1 to Type 5 and Off-Type) were analyzed and prepared in accordance with current legislation. Spectral data were obtained using NIR and hyperspectral measurements covering the VIS/NIR/SWIR regions, and proximate composition was determined for moisture (MOI), starch (ST), protein (PRO), lipids (LIP), fiber (FIB), and ash (ASH). Multivariate analyses and ML classification models were applied to evaluate differences among grain types and commercial categories and to assess the discriminatory capacity of spectral information. The results showed that including physicochemical attributes to evaluate the quality of commercial batches simplifies the commercial categories currently used. For spectral behavior, batches classified as Type 1 and Type 2 showed low reflectance in the NIR and SWIR regions, suggesting greater interaction of radiant energy with compounds associated with nutritional and sensory quality. The MLP, LGBM, CAT, XGB and RF models performed best for the classification of commercial white polished rice batches, with metrics above 95%. The SWIR region, especially the 2173 nm spectral point, demonstrated high discriminatory power. In conclusion, the application of machine learning models based on VIS/NIR/SWIR spectroscopy proved highly efficient for classifying commercial batches of polished white rice, integrating physical and physicochemical attributes of the grains.

## 1. Introduction

Rice (*Oriza sativa* L.) occupies one of the largest areas in global grain production and consumption, and is considered a staple food for the world population due to its nutritional importance and accessibility. The high concentration of starch in its composition makes rice a significant source of energy, in addition to being associated with the prevention of chronic diseases and anti-inflammatory effects [1]. The different processing methods for rice contribute to its versatility, with white polished rice standing out as having the greatest commercial demand [2].

The industrial evaluation of rice quality is directly linked to the physical condition of the grains, although the ideal procedure also encompasses nutritional and sensory parameters. Thus, the industry determines rice quality through physical classification, a processing stage that analyzes physical characteristics and classifies grains into classes and types. The Normative Instructions (No. 06 of 02/2009 and No. 02 of 02/2012) governed by the Ministry of Agriculture, Livestock, and Supply (MAPA) indicate the ideal characteristics, as well as the physical changes considered defects and their respective tolerance levels accepted for commercialization [2,3].

In conventional physical classification, a widely used method in processing units, quality standardization is achieved through visual inspection and manual separation of defects. Due to the way it is conducted, this method has limitations, including excessive demand for qualified labor, excessive execution time, and a propensity to errors [4,5]. The intensive manual activity renders the method interpretive and subjective, making it difficult to impose a single quality standard for commercial batches [3,6].

In this context, indirect technologies emerge as a promising alternative by enabling analyses aligned with the current demands of the food industry. Indirect methods evaluate the quality of materials with agility, precision, and operational simplicity, while allowing the characterization of the internal composition in a non-invasive manner and without causing losses [6,7]. This approach is possible due to the interaction between electromagnetic radiation and the molecular constituents of the sample, facilitating the obtaining of chemical and structural information [3,5,8].

Reflectance spectroscopy is an indirect analysis technique that allows characterization of the proximate composition of grains across the VIS, NIR, and SWIR regions [7,9]. When radiation is incident on a sample, part of the energy is absorbed and another part is reflected, generating differences depending on its composition [5,10]. In reflectance spectroscopy, technologies such as near-infrared (NIR) spectroscopy and hyperspectral (HS) sensors stand out.

The NIR technique has been widely accepted in the literature as an analytical tool, and its applicability has expanded significantly. In rice, it has been used in quality and authenticity control by characterizing the proximate composition of grains [6,11,12,13,14,15]. HS technology, in addition to enabling a more detailed physicochemical characterization, also allows curves to be obtained that reveal the spectral behavior of the grains, expanding the evaluation possibilities [3,16,17,18,19].

The interpretation of these data requires advanced techniques due to the multidimensional nature of the information, such as multivariate analysis and machine learning [20,21]. Multivariate analysis stands out for allowing simultaneous, rapid evaluation, simplifying the analysis of multiple variables, and promoting correlation between spectral data and physicochemical parameters [3], while machine learning (ML) techniques primarily predict continuous and/or categorical variables using supervised algorithms [22,23].

ML algorithms present different modeling strategies and ways of acting. In classification, models are trained to identify patterns in input data and, from there, assign them to classes quickly and efficiently [24]. These algorithms learn decision boundaries from examples, capturing both linear and non-linear relationships that may not be perceptible through conventional analytical methods [25]. In rice grains, predictive classification is adopted to distinguish varieties [14,25,26], morphological [22,24] and sensory [27] characteristics, and commercial batches [3]. As spectral data typically contains many variables and subtle differences associated with chemical composition, ML models become particularly advantageous, as they handle high-dimensional datasets and enhance the detection of discriminatory features.

Therefore, evaluating the quality and classification of rice is an immediate need given the economic importance of the grain, benefiting industry, producers, and consumers. Furthermore, the disadvantages of the traditional method reinforce the need for more efficient and agile classification methodologies that can meet high operational demands with high accuracy. Although NIR and ML approaches have been widely employed in previous studies, these applications generally rely on limited spectral regions, mainly VIS/NIR, and do not incorporate the complementary information available in the SWIR range. The inclusion of these regions broadens the chemical sensitivity of the models, enabling the detection of subtle compositional and structural differences that are not captured by conventional NIR-only approaches. Thus, the present study aimed to characterize the physical and centesimal quality and classify commercial batches of white polished rice using machine learning algorithms based on VIS/NIR/SWIR data.

## 2. Materials and Methods

### 2.1. Study Characterization

**Figure 1:**
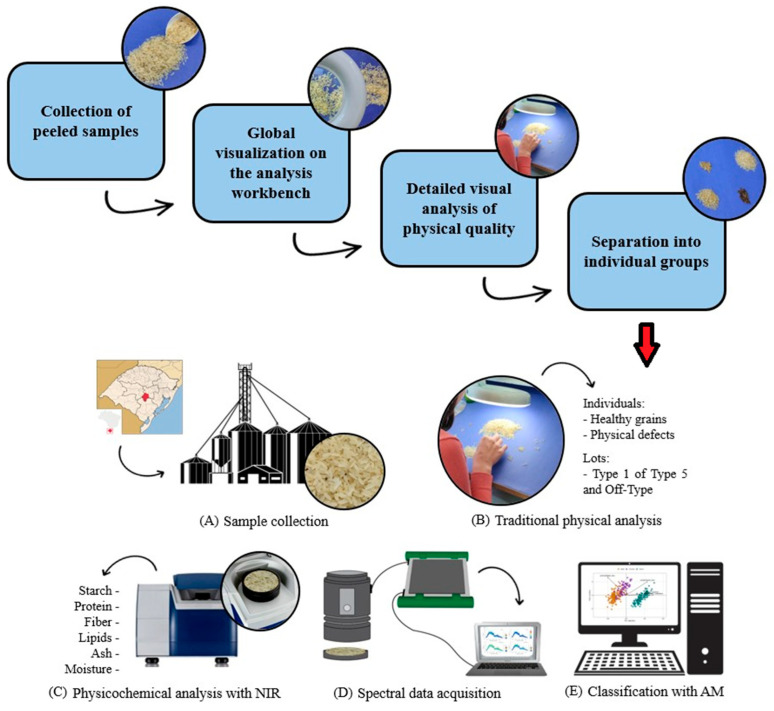
Experimental operations performed: (**A**) sample collection; (**B**) conventional physical analysis; (**C**) compositional analysis using NIR; (**D**) acquisition of spectral curves; (**E**) application of ML algorithms for classification.

The polished white rice samples were provided by Indústria de Alimentos Treichel^®^, located in Cachoeira do Sul, Brazil, under the geographic coordinates 30°0′45″ S (latitude), 52°55′11″ W (longitude), and 73 m above sea level, in the Rio Grande do Sul. The collection was conducted at the mill during grain processing, immediately after hulling, during the 2022/23 harvest. Samples were collected from a single processing unit. The study was conducted as follows as represented in Figure 1.

The analyses were conducted at the Post-Harvest Laboratory (LAPOS), located at the Universidade Federal de Santa Maria–Cachoeira do Sul Campus, in association with the Physicochemical Analysis Laboratory, located at the Food Research Center (CEPA) of the Universidade de Passo Fundo, in the city of Passo Fundo, and the Spectroscopy Laboratory of the Universidade Federal do Mato Grosso do Sul–Chapadão do Sul Campus (UFMS-CS).

### 2.2. Conventional Physical Analysis

The classification was carried out using the conventional method, in accordance with Normative Instructions No. 06 of 02/16/2009 and No. 02 of 02/07/2012 (MAPA), at the Post-Harvest Laboratory (LAPOS) at the Universidade Federal de Santa Maria–Cachoeira do Sul Campus. The materials used for the physical evaluation were: blue sulphite cardboard paper, attached to the classification bench to contrast the color of the grains; a magnifying glass with a light to help identify the characteristics; and tweezers to move the grains.

**Figure 2:**
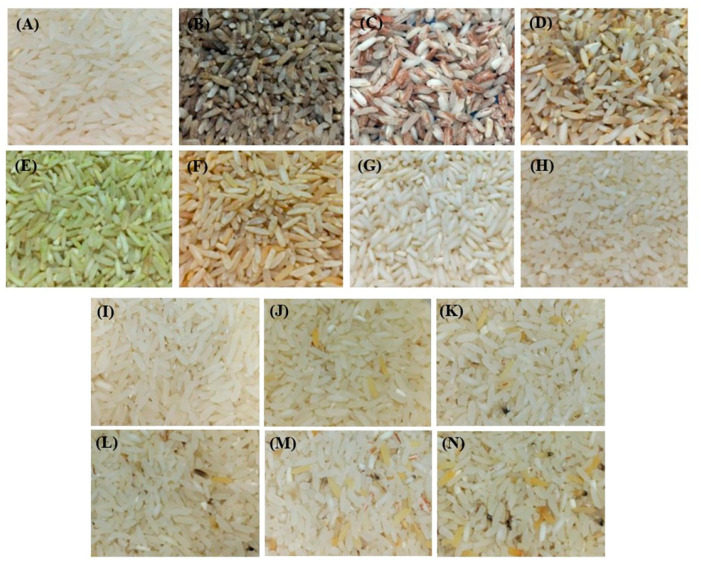
Individual samples of physical defects and samples of commercial lots of polished white rice grains: (**A**) healthy grains, (**B**) burnt, (**C**) streaked, (**D**) pitted or spotted, (**E**) green, (**F**) yellow, (**G**) chalky, (**H**) broken, (**I**) Type 1, (**J**) Type 2, (**K**) Type 3, (**L**) Type 4, (**M**) Type 5, (**N**) Off-Type.

The grains were placed on the bench, and their physical quality was visually examined. The grains with physical changes were separated and classified according to the characteristics of each identified defect. In cases where more than one defect was found in a single grain, the classification was based on the defect considered most serious by the standard. The physical defects found in white polished rice were: healthy grains, burnt, streaked, pitted or spotted, green, yellow, chalky, and broken (Figure 2A–H). The healthy grains constituted an individual sample free from defects and were included in the composition of the commercial batch samples.

According to MAPA regulations, the commercial classification of polished rice is based on the proportion of defective grains in the sample, which determines its placement into one of five official types (Type 1 to Type 5). Type 1 represents the highest quality, allowing only a minimal percentage of defective grains, while Types 2, 3, and 4 progressively permit larger defect proportions. Type 5 corresponds to the lowest commercial grade, characterized by the highest allowable percentage of defective grains. Samples that exceed the maximum defect limits established for Type 5 are classified as Off-Type indicating that they do not meet the minimum quality standards for commercial grading. Thus, the types are determined by the cumulative percentage of each defect category identified during the physical evaluation (Figure 2I–N).

### 2.3. Sample Preparation

After identifying the defects, the samples were divided as follows: (i) physical defects and healthy grains, and (ii) commercial batches from Type 1 to Type 5 and Off-Type (Figure 2). To characterize individual physical defects and healthy grains, 100 g samples were separated and ground in a knife mill (20–30 mesh sieve) to produce a homogeneous fine powder. The samples from commercial batches were prepared according to the percentages permitted by current legislation. From this classification, 2 kg samples were prepared, subdivided into 20 g fractions, yielding 100 subsamples with grains in their original form.

### 2.4. Indirect Physicochemical Analysis

The physicochemical analysis was conducted at the Physicochemical Analysis Laboratory of the Food Research Center (CEPA) at the Universidade de Passo Fundo, using an indirect approach based on near-infrared spectroscopy (NIR). For the analytical procedure, a high-precision DS2500 spectrometer (FOSS, Hillerød, Denmark) was used. The proximate composition of individual samples (physical defects and healthy grains) and commercial batches (Type 1 to Type 5 and Off-Type) was quantified in triplicate, consisting of starch (ST), protein (PRO), moisture (MOI), lipids (LIP), fiber (FIB), and ash (ASH).

The homogenized samples were transferred to the equipment’s capsule. The equipment emits a specific beam of near-infrared light, causing the molecules that form the material’s chemical bonds to vibrate. From this, the energy is absorbed and/or reflected by the sample, depending on the grain composition. The final measurement corresponds to the difference between the emitted and the reflected energy detected by the instrument, generating a unique spectrum for each sample. Spectral data were collected in reflectance mode over the 400–2500 nm range.

#### Statistical and Multivariate Analysis of Physicochemical Parameters

The data set resulting from the VIS/NIR techniques was initially subjected to analysis of variance (ANOVA) using the F test to identify significant differences between individual treatments and commercial batches. Before applying ANOVA, the assumptions of normality and homoscedasticity were verified. Normality of residuals was evaluated using the Shapiro–Wilk test, and homogeneity of variances was assessed using Levene’s test, both performed in RStudio (version 4.3.3). The means were grouped using the Scott-Knott test at the 5% significance level, using the Sisvar 5.8 software [28]. The Pearson correlation network was also constructed using the Rbio software, version 1.9.2 [29]. In the network, edge thickness was adjusted using a cutoff value of 0.60 to highlight connections. Positive correlations (0 to 1) were shown in green, whereas negative correlations (−1 to 0) were shown in red.

Principal component analysis (PCA) was employed to reduce the dimensionality of the dataset. This analysis is a multivariate statistical technique that reveals hidden patterns and possible groupings. This method converts the original variables into principal components, defined as linear combinations that summarize the variability present in the dataset [20]. PCA was performed using RStudio (version 4.3.3) and the libraries factoextra, FactoMineR, and ggplot2 for data processing and visualization.

### 2.5. Hyperspectral Data Collection

**Figure 3:**
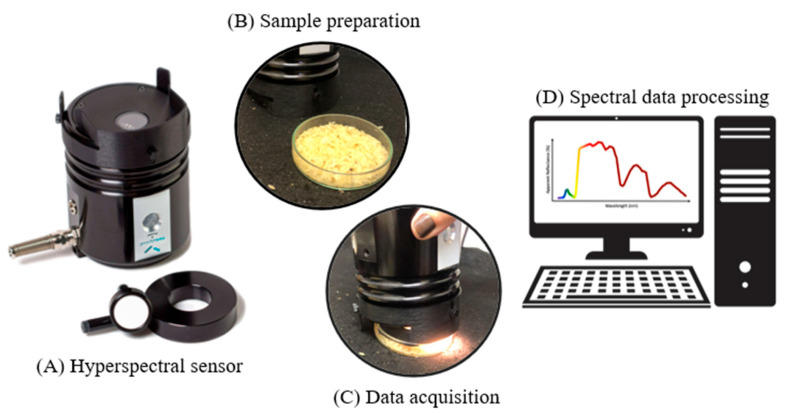
Acquisition of spectral data from batches of white polished rice using a hyperspectral sensor.

Hyperspectral reflectance data were recorded for commercial batches (Type 1 to Type 5 and Off-Type) using a FieldSpec 4 Jr spectroradiometer (Analytical Spectral Devices, Boulder, CO, USA) equipped with a muglight, operating across the 350–2500 nm range. The spectral resolution is 3 nm from 350 to 700 nm and 30 nm from 1400 to 2100 nm, with sampling intervals of 1.4 nm from 50 to 1050 nm and 2 nm from 1000 to 2500 nm. Besides operational benefits, this approach avoids interference from ambient light, thereby reducing errors associated with stray light (Figure 3).

A white barium sulfate plate, which reflects all incident light (100%), was employed as the reference standard. The spectral data from the plate were measured and used to determine the reflectance factor, which was applied to each samples reading. For reading, the samples were arranged in an 8 cm Petri dish, from which three consecutive readings were obtained; the mean was subsequently determined. Data were recorded on a computer using sensor-specific software (RS^3^) version 4.0. The generated files were imported into ViewSpectroPro version 6.0, which was used to export the data in (.txt) format.

### 2.6. Analysis of Spectral Behavior

Spectral data analysis over 400–2500 nm was conducted in Python 3.10. The spectral range from 350 to 400 nm was disregarded in the analysis as it was considered noise. To construct mean spectral signatures per class, the matplotlib library was used to highlight common patterns and differences. Additionally, individual signatures were generated in 3D using the Axes3D module to assess the internal variability of samples within each class. Within the scope of multivariate analysis, principal component analysis (PCA) and linear discriminant analysis (LDA) were used, both implemented with functions from the scikit-learn library. PCA was preceded by data standardization using StandardScaler. As an alternative to the restricted use of only two principal components, which could lead to substantial loss of discriminative information, it was decided to retain 50 principal components to balance dimensionality reduction with the preservation of available spectral richness.

To support the interpretation of the hyperspectral information, it is important to note that the VIS/NIR/SWIR regions contain absorption features associated with specific chemical bonds present in the main compositional constituents of polished white rice. In the VIS region (400–700 nm), reflectance is mainly influenced by surface scattering and residual pigments [3]. In the NIR and SWIR regions (700–2500 nm), the most relevant absorption features arise from overtone and combination bands of fundamental vibrations related to O–H, C–H, and N–H bonds [3,15]. These bonds are directly associated with moisture (O–H stretching), starch (C–H and O–H groups), proteins (N–H and C–H groups), and lipids (C–H stretching) [9,27]. Additional SWIR absorption features arise from stronger combination bands involving C–H and O–H groups, contributing to the differentiation of starch, lipids, and moisture [9,15,27].

### 2.7. Machine Learning for Classification

In the supervised modeling stage, the panda’s library was used to read and organize tables into matrix structures, as well as to process columns, select numerical bands corresponding to wavelengths, and remove inconsistencies in class labels. Data standardization was performed using the StandardScaler function from the scikit-learn library, ensuring that all variables had a mean of 0 and a standard deviation of 1. Any missing values were imputed using the mean of the training set, avoiding information leakage between cross-validation subsets.

The dataset consisted of 600 samples, uniformly distributed across the six commercial rice classes included in the study: Off-Type (100 samples), Type 1 (100), Type 2 (100), Type 3 (100), Type 4 (100) and Type 5 (100). This naturally balanced distribution made additional resampling or class-balancing techniques unnecessary during model training.

From this, eight classification algorithms were evaluated (Table 1): Random Forest (RF), Gradient Boosting (GB), Support Vector Machine (SVM), K-Nearest Neighbors (KNN), Multilayer Perceptron (MLP), Xtreme Gradient Boosting (XBG), Light Gradient Boosting Machine (LGBM), and Categorical Boosting (CAT); and a traditional regression model: Logistic Regressor (LR). The RF, GB, SVM, KNN, MLP, and LR models were obtained directly from the scikit-learn library, while XGB, LGBM, and CAT were accessed via the xgboost, lightgbm, and catboost libraries, respectively. Each model was configured with random_state = 42 to ensure reproducible results. Validation was performed through cross-validation with KFold of 10 subdivisions (9 parts for training and 1 part for testing), ensuring greater statistical robustness.

The rice classes presented a naturally balanced distribution, so no additional resampling techniques were necessary. The risk of overfitting was mitigated by using stratified cross-validation, together with the regularization of mechanisms already inherent to the algorithms and by ensuring that all preprocessing steps were applied exclusively to the training folds.

To ensure methodological transparency, all hyperparameters used in the configuration of each model are summarized in Table 2. These settings follow values commonly adopted in studies involving spectral data in agricultural sciences and were kept consistent across folds to guarantee reproducibility.

Classifier performance was evaluated based on multiclass metrics, including accuracy, precision, recall, and F1-score. These metrics were derived from the cross-validation results and calculated using scikit-learn functions. The results were compared using boxplots to visualize the variability between validation folds. To evaluate whether the differences among algorithms were statistically significant, an analysis of variance (ANOVA) was applied, complemented by the Scott-Knott cluster test at 5% probability, implemented in the Sisvar software version 5.0, grouping the models into different letters according to their performance in each metric. Finally, the most representative spectral bands were selected for classification. This step used the importance of variables derived from the Random Forest algorithm (*feature_importances*).

The feature importance values obtained from the Random Forest classifier correspond to impurity-based (Gini) importance. These scores are normalized by the algorithm so that all feature importances sum to 1.

## 3. Results and Discussion

### 3.1. Proximate Characterization of White Polished Rice

The results of the analysis of variance and mean test for the proximate composition of physical defects and healthy grains of white polished rice are presented in Table 3. A statistical difference (*p* ≤ 0.05) was found between the treatments and all physicochemical variables analyzed in the study. The evaluated grains presented a mean moisture content of 12.52%, a value compatible with the international standard for preserving the quality of milled rice, established by the Codex Alimentarius committee (CXS 198-1995), which recommends a maximum moisture content of 14% [35]. The highest moisture content was observed in chipped or stained, broken, and healthy rice grains, at 12.74%, 12.73%, and 12.71%, respectively.

The starch content was higher in healthy (66.64%) and broken (66.65%) grains. Even with an inferior structure, the broken grains maintain a significant amount of starch, which helps to explain the greater participation of this physical defect in the composition of Type 1. The streaked grains presented the lowest starch content among the defects studied, being 65.38%. The origin of this defect is associated with climatic variations throughout cultivation and inadequate storage conditions, which cause microcracks and opacity in the grains [36]. According to [2], there is a lower density of starch granules and more air spaces in opaque regions.

Regarding protein content, green grains had the highest (12.57%), followed by heat-damaged grains (10.29%). According to [37], incomplete maturation in green grains limits starch accumulation and favors protein deposition, as the aleurone tissue develops earlier than the endosperm. In heat-damaged grains, a defect resulting from enzymatic activity during post-harvest handling, the starch content is partially degraded by the enzymes, which explains the lower starch content (60.95%) and, consequently, the greater accumulation of protein [3].

The highest lipid contents were observed in yellow (2.82%) and green (2.74%) grains. In yellow grains, this increase is related to inadequate drying and storage conditions, which can stimulate the release of free fatty acids, especially in the presence of moisture, increasing the lipid fraction in relation to the total mass of the grain [38]. In green grains, the faster development of naturally lipid-rich regions, such as the germ and aleurone layer, contributes to greater lipid deposits [39].

The fibers showed the greatest variation among the physical defects evaluated in the study, with emphasis on the heat-damaged grains, which recorded the highest concentration (2.10%). According to [40], the outer layer of these grains tends to present greater resistance to abrasion during processing, promoting the preservation of fiber content even after the whitening and polishing stages. Furthermore, ref. [41] highlighted that small changes in the peripheral layers of processed grains can significantly impact the fiber content, since the constituent fraction is predominantly concentrated in this region.

The heat-damaged (1.81%) and green (1.79%) grains had the highest ash content. This increase can be attributed to the greater preservation of the outer layers in both after processing, which concentrates the mineral content of the rice [3]. These physical defects are also linked to the lower development of the endosperm in relation to the bran and germ, which proportionally increases the participation of ash in the grain structure. In contrast, healthy (1.34%) and broken (1.38%) grains exhibited the lowest ash percentages, indicating lower micronutrient concentrations.

**Figure 4:**
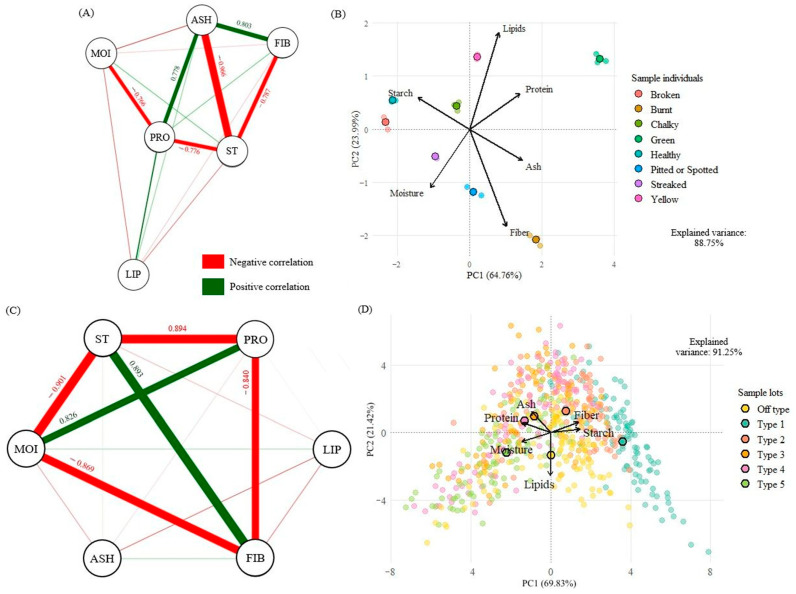
Pearson correlation network between the centesimal composition variables of individual samples of physical defects and healthy grains of polished white rice (**A**): Moisture (MOI), Starch (ST), Protein (PRO), Lipids (LIP), Fiber (FIB), Ash (ASH); PCA of the physicochemical components of individual samples of physical defects and healthy grains of polished white rice (**B**); Pearson correlation network between the centesimal variables of commercial lots of polished white rice (**C**): Moisture (MOI), Starch (ST), Protein (PRO), Lipids (LIP), Fiber (FIB), Ash (ASH); PCA of the physicochemical properties of commercial lots (Type 1 to Type 5 and Off-Type) of polished white rice (**D**).

Figure 4A presents the Pearson correlation network between the physicochemical variables of healthy grains and individual physical defects. A strong negative correlation was identified between ASH and ST (r = −0.966), as well as moderate positive correlations between ASH and FIB (r = 0.803) and ASH and PRO (r = 0.778). These relationships indicate that, individually, grains with a higher ash content tend to have a lower starch content and higher concentrations of fiber and protein. This pattern may be associated with the preservation of peripheral layers in grains with physical alterations [36] or with a high degree of polishing, which favors the predominance of starch over constituents external to the endosperm [2].

Furthermore, reduced starch concentrations contribute to an increase in the relative amounts of other constituents in the grain. In line with this, mean negative correlations were observed between ST and FIB (r = −0.787) and between ST and PRO (r = −0.776). Similar behavior was reported by [42] when evaluating the centesimal composition and digestibility of starch in rice varieties, which showed contrary correlations exceeding r = −0.70. Furthermore, although the study focuses on the structure of starch, ref. [43] highlighted that higher starch levels are associated with greater digestibility, indicating lower concentrations of components such as fiber and protein.

A medium negative interaction was identified between MOI and PRO (r = −0.766). According to [15], excess moisture contributes to the solubilization of proteins present in rice grains. Hence, the presence of water in the grain structure tends to reduce the formation of solid compounds, such as proteins, which may also explain the inverse relationship between these variables [44]. Figure 4B below shows the PCA of the physicochemical properties of physical defects and healthy grains of white polished rice.

The first principal component (PC1) accounted for 64.76% of the total variance and showed a strong association with fiber, moisture, and starch. The second principal component (PC2) accounted for 23.99% of the variance and was mainly related to lipids and proteins. The vector arrangement showed a positive correlation between fibers and ash, while both showed negative correlations with moisture and starch, with opposite directions. These relationships were highlighted in the studies by [41,43], which showed that increases in fiber and ash were not accompanied by increases in starch content in rice grains. On the second axis of variation, lipids and proteins showed a positive correlation.

Regarding the distribution of physical defects, the heat-damaged and green grains were positioned close to the fiber, protein, ash, and lipid vectors, suggesting a greater concentration of these constituents. Such physical defects are associated with the preservation and early development of the peripheral layers, in which these compounds predominate [3]. On the other hand, healthy and broken grains remained grouped toward the starch vector, indicating its predominance in the composition of these batches. According to [45], the complete development of the endosperm in healthy grains favors the accumulation of starch.

The chalky and yellow grains were concentrated in the central region of the analysis, close to the lipid and protein vectors, indicating a partial influence of these variables [46]. observed that chalky grains may present a higher proportion of lipids in the endosperm compared to translucent grains. Furthermore, according to [38], the release of free fatty acids in yellow grains, caused by inadequate processing conditions, contributes to the increase in the lipid fraction. The streaked, chipped, or stained grains showed an intermediate composition when grouped near the center, suggesting a balanced influence among the analyzed variables.

### 3.2. Commercial Batches

Table 4 presents the analysis of variance and mean comparison test for the proximate composition of samples from Type 1 to Type 5 and Off-Type. The results indicated statistically significant differences (*p* ≤ 0.05) between the batches and the investigated variables. The highest moisture percentage was observed for Type 5 (13.06%), which, although high, remained below 14%, as determined by the international quality standard for milled rice [35]. Despite the variability caused by grain non-uniformity, Types 3, 4, and Off-Type showed similarity among themselves, suggesting a possible grouping based on moisture percentage. Types 1 (73.39%) and 2 (71.65%) had the highest starch contents, while Types 4 (70.09%) and 5 (69.24%) had the lowest. According to [47], starch is directly related to the sensory characteristics that denote quality. The decrease in starch content in batches with lower physical quality may be associated with a greater number of defects, in which other compounds predominate [48]. Starch contents did not differ significantly between Type 3 and Off-Type, revealing that both have a similar concentration of this constituent.

Type 1 had the lowest protein content (8.05%), suggesting that higher physical quality is associated with lower protein content. Refs. [13,44] highlight that increasing starch content can reduce protein concentration, due to changes in the distribution and proportions of solid compounds in the grain. In contrast, the highest protein contents were observed in Types 3 (9.31%), 4 (9.47%), and 5 (9.57%), which consisted mainly of green and heat-damaged grains, with no significant differences among them. Taken together, these results demonstrate that classifying rice quality based on protein content tends to reduce the number of commercial categories.

The Off-Type classification presented the highest lipid content (1.71%). Refs. [2,39] reported that lipid content is generally higher in grains with physical defects, due to fragmentation of the outer layer during processing. According to ref. [49], the increase in lipid content, especially in the presence of moisture, intensifies oxidative processes that can generate unpleasant flavors. Types 2 (1.43%), 3 (1.45%), and 4 (1.45%) had the lowest lipid levels, with no statistical difference between them. This pattern shows that these categories are equivalent in lipid content; thus, a single class could represent them for this attribute.

Fiber contents varied significantly between batches, with the highest content recorded in Type 1 (2.10%) and the lowest in Type 5 (1.86%). The increase in better physical quality classifications can be justified by the presence of whole, well-formed grains in Type 1, which preserve the fibrous structure during processing [3]. Although isolated defects may have a higher fiber content, the overall composition of the sample may reflect a lower mean fiber content in batches of lower physical quality. Type 3 and Off-Type were statistically equivalent, indicating no distinction between the fiber content classes.

The ash content showed low variation between classifications, with the lowest content observed in Type 1 (1.12%). For this constituent, Types 2 (1.18%), 3 (1.19%), 4 (1.18%), and Off-Type (1.18%) belong to the same statistical group, which can be used to summarize the batches by ash content, since it does not differ between them. This constituent is directly associated with the peripheral layers of the grain and the concentration of essential minerals such as phosphorus, potassium, magnesium, iron, and zinc [2]. According to ref. [6], lower ash content indicates more polished grains.

Figure 4C presents the Pearson correlation network for the proximate composition of the types of white polished rice (Type 1 to Type 5 and Off-Type). A strong positive correlation was observed between ST and FIB (r = 0.893), indicating that both variables tend to increase or decrease together in commercial batches. This behavior may be associated with genetic factors, the presence of grains with a lower degree of polishing, or even the occurrence of soluble fiber fractions in the endosperm [2,50]. Ref. [42] highlight that both constituents play important roles in vital activities and contribute to the batch’s nutritional quality. MOI and PRO showed a strong positive correlation (r = 0.826), suggesting a direct relationship between them. In this context, commercial types with higher moisture content tend to have higher protein content. Ref. [3] reported a similar result in parboiled rice grains, attributing this behavior to the hygroscopic capacity of proteins. Complementarily, ref. [51] identified the same relationship between the variables, suggesting that certain moisture ranges may favor the accumulation of protein in rice grains, especially when higher.

On the other hand, MOI showed strong negative correlations with ST (r = −0.901) and FIB (r = −0.869). This relationship may be associated with the proportion of constituents, since increasing water content tends to reduce solids concentration, as described by [52]. Therefore, grades with higher moisture content tend to have lower starch content, as is the case with Type 5. Ref. [53] confirmed that high moisture levels significantly decrease the concentration of this component. Ref. [51] highlight that the structural nature of fibers, predominantly insoluble, contributes to more fibrous grains having a lower water absorption capacity.

Inverse relationships were also identified between PRO and ST (r = −0.894) and between PRO and FIB (r = −0.840), evidenced by strong negative correlations between the variables. In this sense, classifications with higher protein contents tend to have low concentrations of starch and fiber. Although unusual, this behavior between proteins and fibers can be justified by genetic factors, cultivation conditions, and processing levels [2,43]. Ref. [3] point out that physical classifications considered to be of low quality may present a higher protein content due to the large presence of physical defects, which, individually, present a predominance of this compound.

Figure 4D shows the PCA of the proximate composition of commercial batches of polished white rice (Type 1 to Type 5 and Off-Type). The first principal component (PC1) explained most of the total variance (68.99%), distinguishing grains with higher starch and fiber contents from those with higher moisture and lipid contents. The second principal component (PC2) accounted for 22.50% of the variation, separating batches with high protein and ash concentrations from those with lower levels of these components. The analysis also identified a strong positive correlation between starch and fiber, while both showed negative correlations with moisture and lipids. This behavior corroborates the findings highlighted by Pearson’s correlation network.

Regarding batch grouping, Type 1 showed a strong association with starch, suggesting higher concentrations of this component. This result corroborates the high starch means found for whole and individually broken grains, which mostly make up this classification. Type 2 showed a stronger correlation with fiber content, while Type 3 showed a stronger association with protein and ash. Type 4, in turn, showed a significant relationship with proteins, reinforcing the tendency for variation in this component across batches.

Type 5 was positioned close to the moisture vector, suggesting that it has a greater water-retention capacity. However, water content is strongly influenced by batch and processing methods, potentially compromising its representativeness for batch characterization [3]. The Off-Type classification demonstrated proximity to the lipid vector, evidencing that this component constitutes a distinctive characteristic of the category. In general, principal component analysis (PCA) revealed marked differences between the classifications, reinforcing the possibility of distinguishing between commercial batches based on their proximate composition.

Current legislation establishes six commercial categories of rice, defined by physical quality criteria. However, the analysis of the percentage means of the physicochemical constituents of the evaluated batches revealed significant similarities between certain classes, indicating that some physical differences do not reflect substantial differences in nutritional attributes. This result suggests that, from a physicochemical perspective, current categories could be unified. Thus, classification based on physicochemical evaluation tends to reduce the effective number of commercial categories for each attribute analyzed.

These findings also indicate that centesimal composition can serve as a more strategic basis for segregation, allowing batches to be directed according to their predominant nutritional constituents rather than their physical characteristics. By relating specific physicochemical attributes to technological performance, the results show that raw materials can be allocated more efficiently to particular industrial uses, such as processes that benefit from higher protein content or greater starch availability. This targeted allocation diminishes the need for numerous commercial classes and avoids the time-consuming segregation of batches that share equivalent nutritional profiles, thereby reinforcing the potential of physicochemical profiling to support a more rational and streamlined classification system.

### 3.3. Spectral Behavior of Commercial Batches of Polished White Rice

**Figure 5:**
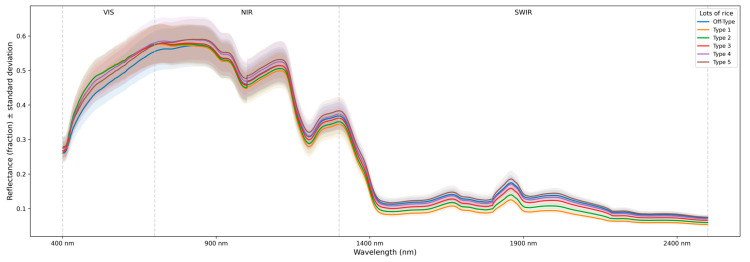
Average spectral signatures for commercial lots of polished white rice (Type 1 to Type 5 and Off-Type).

The spectral means of each commercial batch analyzed are presented in Figure 5. Although all batches exhibited a similar overall pattern, substantial differences were observed in specific spectral bands. The commercial batches did not show any evident distinction in the initial visible region (400–500 nm). However, from 500 nm onwards, discrepancies became noticeable, mainly in the NIR and SWIR bands. Previous studies conducted by ref. [54] reported analogous behavior in rice grains in the visible region, while refs. [3,55] highlighted striking differences in the NIR and SWIR bands.

The batch classified as Off-Type showed the lowest reflectance in the 500–700 nm range, indicating greater absorption of visible light. According to ref. [3], this pattern is associated with the presence of pigments in grains with physical defects, which are predominant in this batch. Consistently, ref. [56] observed that damaged rice grains exhibit greater absorption variability in the visible (RGB) range. In the same vein, ref. [57] highlighted that attributes such as color, pigmentation, texture, and brightness directly influence the spectral behavior of grains.

Still in the VIS region, batches Types 1, 2, and 3 presented the highest reflectances, resulting from the predominance of intact and well-formed grains [3]. Ref. [58] highlighted that translucent grains, with a more organized structure, favor a more uniform interaction with light. In the NIR, a sharp drop in reflectance was observed for all batches, attributed to the interaction of radiation with chemical groups [34]. In SWIR, the reduction was even more significant, also due to interactions with molecular bonds (C-H, N-H, and O-H), associated with the content of water, starch, and proteins [15,52].

Types 4, 5, and Off-Type showed the highest reflectances in the NIR and SWIR. This pattern suggests less interaction between radiation and chemical bonds, possibly reflecting nutritional inferiority, since absorption in the near-infrared is related to the physicochemical attributes of the grains [59]. In contrast, Type 1 exhibited the lowest reflectances in these regions, indicating greater interaction of radiation with its chemical bonds. Ref. [60] highlight that well-structured starches tend to absorb radiation, particularly in the near-infrared, which explains the low reflectance in this batch.

The drop in reflectance from 1900 nm onwards reinforces the strong association of SWIR with compounds such as water, starch, and proteins. Ref. [11] reported that the region near 2110 nm correlated with protein content, and that the peak at 1933 nm correlated with amylose in white rice. Similarly, ref. [59] highlighted the range between 2288 and 2369 nm as indicative of proteins in white rice, while ref. [15] reported protein overtones between 2469 and 1509 nm. Water is also strongly associated with specific SWIR peaks. Ref. [61] observed the interaction of radiation with water molecules at 1940 nm in white and paddy rice, and ref. [55] identified the peak at 1855 nm in paddy rice.

**Figure 6:**
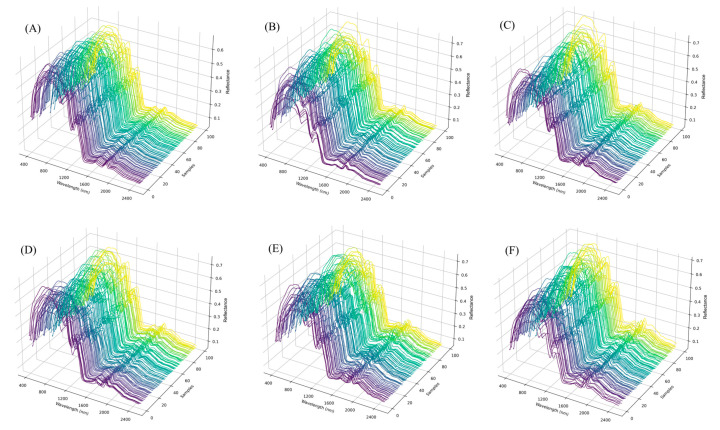
Spectral signatures (3D) by samples for each type of white polished rice: Type 1 (**A**), Type 2 (**B**), Type 3 (**C**), Type 4 (**D**), Type 5 (**E**), and Off-Type (**F**).

Figure 6A–F displays the 3D spectral signatures for each commercial batch of white polished rice. Greater variation in spectral behavior was observed among samples of Types 2, 4, and 5, indicating greater heterogeneity in the physical and centesimal composition of the grains that comprise these batches. In contrast, Type 1 demonstrated high uniformity among samples, suggesting greater structural and compositional similarity. According to ref. [3], this pattern is associated with the grouping of well-developed grains and the lower occurrence of physical defects.

**Figure 7:**
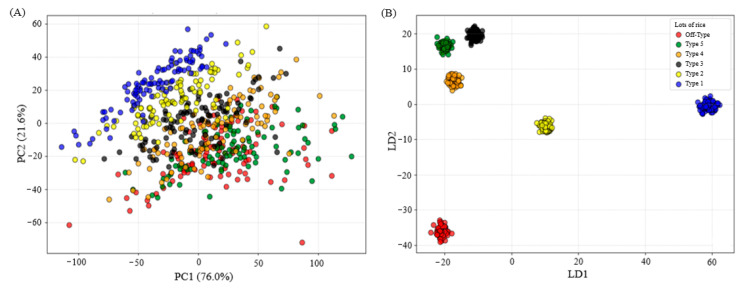
PCA (**A**) and LDA (**B**) for spectral data of commercial lots of white polished rice (Type 1 to Type 5 and Off-Type).

Figure 7 presents the exploratory analyses, conducted using PCA and LDA, based on the spectral data of commercial batches of polished white rice. In the PCA (Figure 7A), a highlighted grouping of Type 1 was observed, indicating that the grains in this batch have well-defined, distinct characteristics compared to the others. This result corroborates the behavior evidenced in the spectral curves, reinforcing the homogeneity of the samples. The physical classification of Type 1 concentrates grains with superior characteristics, including good formation, structural organization, and absence of pigmentation [2,3].

The two-dimensional projection also revealed partial overlap for Type 2 and more pronounced overlap for Types 3, 4, 5, and Off-Type. This result suggests that although there is relevant spectral variation between the samples, the natural separation between the classes is not fully perceptible from the global variance of the data alone. The strong overlap observed between classes results from the high spectral correlation between the bands, which makes spatial separation difficult. Furthermore, the gradual presence of physical defects in batches of lower physical quality, combined with similarities in certain physicochemical properties, may contribute to the overlap of these groups [60].

This finding reinforces the need for methods that explicitly account for class information to improve discrimination between groups. In this context, the LDA (Figure 7B) showed clear separation between the commercial rice batches, resulting in well-defined groups. This result indicates that, when class information is considered, linear combinations of spectral bands maximize intergroup separation while minimizing intragroup dispersion, highlighting the discriminatory potential of spectral signatures.

### 3.4. Classification of Commercial Batches of Polished White Rice Using Machine Learning

**Figure 8:**
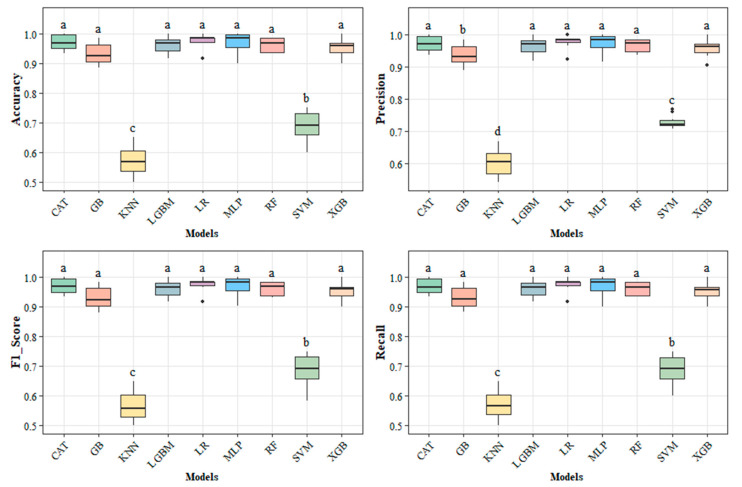
Boxplots for the performance means of each predictive model used in the classification of commercial lots of polished white rice. Means with the same letters do not differ from each other by the Scott-Knott test at 5% probability.

Figure 8 presents the performance metrics for the predictive models used to classify commercial batches of polished white rice. The results showed statistically significant differences (*p* ≤ 0.05) between the models evaluated. The Multilayer Perceptron (MLP) algorithm stood out by achieving 97.2% accuracy and 97.5% precision, with performance closely matching that of the Logistic Regressor (LR) model used as a comparative reference. These findings reinforce the effectiveness of artificial neural networks, such as MLP, in classifying rice grains.

Previous studies corroborate these results. Ref. [62] reported a mean accuracy of 99.46% in the classification of five rice varieties using MLP networks, considering all the morphological and chromatic characteristics of the grains. By selecting only features deemed relevant for classification and reducing the input set, the algorithm maintained a high accuracy of 98.48%. Similarly, ref. [24] observed accuracies above 99% when classifying rice varieties with MLP. More recently, ref. [32] implemented a hybrid approach combining MLPs and XGB, integrating neural networks and decision tree boosting, achieving an accuracy of 99.86% in multiclass rice variety classification.

Even with the highest metrics to MLP, decision tree-based ensemble and random forest models also demonstrated statistical equivalence and impressive performance. The Categorical Boosting (CAT), Light Gradient Boosting Machine (LGBM), Random Forest (RF), and Xtreme Gradient Boosting (XGB) algorithms achieved metrics above 96%, with no statistically significant differences between them. These results are superior to those reported in the literature for some models, which can be attributed to the high discriminatory power of the data set, thereby maximizing predictive effectiveness [25]. Ref. [34] achieved 91.24% accuracy with the CAT algorithm and 91.07% with XGB in classifying rice varieties.

Although the literature reports satisfactory performance of the SVM model in grain classification [3,27,31], in this study, the algorithm achieved an accuracy of 68%, lower than that of neural networks and boosting algorithms. The lowest performance was observed for KNN, which achieved 56% accuracy in categorizing commercial rice batches. Given the performance, neither model is very suitable for classifying commercial batches of polished white rice. It is worth noting that, according to [3], given the complexity of the multiclass task, only metrics above 95% indicate excellence and robustness in predictive models.

A growing number of studies have explored deep learning and hybrid approaches to improve predictive performance in rice quality assessment. The DCGAN-based framework proposed in ref. [18] enhanced hyperspectral modeling by augmenting data and strengthening spectral feature extraction. Hybrid strategies have also shown promise: ref. [32] reported performance gains in multiclass classification through the combination of deep neural networks with imbalance-aware learning, while ref. [45] demonstrated that integrating CNNs with boosting improves prediction accuracy compared with conventional spectral models. Although these methods achieve high accuracy, they typically require larger datasets, greater computational resources, and more complex training pipelines. In contrast, the MLP, RF and boosting models evaluated here reached competitive performance using only spectral information, reinforcing their practicality for industrial applications.

**Figure 9:**
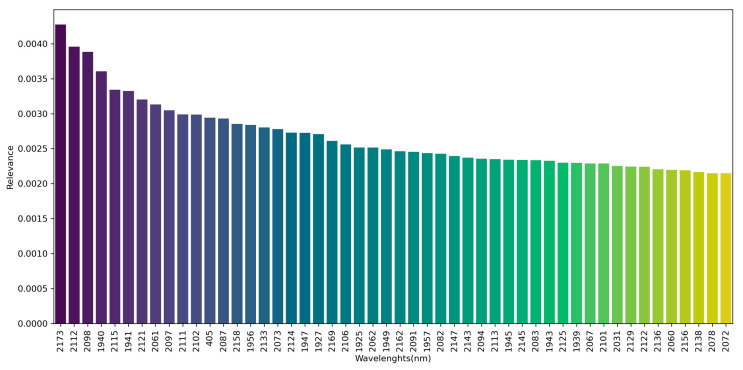
Fifty most representative spectral points for the classification of commercial batches of polished white rice.

Figure 9 demonstrates the fifty most representative spectral bands for the classification of commercial batches of polished white rice. Greater relevance was observed for wavelengths in the SWIR region, which exhibited greater discriminatory weight among commercial batches of polished white rice. The range from 1900 to 2200 nm contained the largest number of features considered relevant to the classification performance of the predictive models, with significant emphasis on the 2173 nm.

The literature associates these wavelengths with properties related to proximate composition, especially the presence of protein groups [11,15] and water content [52,55]. According to ref. [63], this region is strongly influenced by specific chemical bonds, which explains the sensitivity to representative molecular constituents in grains. The identification of these bands confirms the potential of hyperspectral sensors to capture relevant physicochemical information, demonstrating their applicability as a basis for robust predictive models to discriminate commercial batches of polished white rice.

In addition, in the VIS region, the spectral point at 405 nm demonstrated discriminatory relevance. Consistently, ref. [64] noted the region from 400 nm as particularly informative for discriminating pigments in rice grains, possibly due to the interaction of radiation with phenolic compounds (phenols and flavonoids) associated with coloration. Likewise, ref. [3] identified the 350–750 nm range as the most relevant for discriminating characteristics associated with parboiled rice classification. This region, as noted by ref. [64], is highly sensitive to changes in rice processing, such as polishing, which alters the grain’s surface composition and visual properties.

### 3.5. Limitations and Future Research

This study presents limitations that should be considered when interpreting the results. All samples were obtained from a single rice milling unit and correspond to a single harvest season, which restricts the geographic and temporal representativeness of the dataset. Although different commercial categories and off-type grains were included, this limited scope may reduce the applicability of the findings to broader production systems, distinct cultivars, variable environmental conditions, or alternative processing protocols.

In addition, although hyperspectral data combined with machine learning demonstrated strong predictive potential, the study did not evaluate practical aspects related to industrial implementation, such as acquisition cost, infrastructure requirements, computational demands, and compatibility with existing sorting equipment. These factors may influence the feasibility and scalability of technological adoption.

Future research should therefore expand sampling across different milling units, regions, cultivars, and harvest years, and incorporate a broader range of physical and physicochemical variability to enhance models robustness and generalization. Additionally, techno-economic analyses and pilot-scale evaluations are needed to assess cost–benefit relationships, processing throughput, and operational constraints, providing a more comprehensive understanding of the industrial viability of hyperspectral-based classification systems.

## 4. Conclusions

The application of predictive machine learning models based on VIS/NIR/SWIR spectral regions demonstrated high efficiency in classifying commercial batches of polished white rice, accounting for the grains’ physical and physicochemical attributes. Thus, it is concluded that: (i) there is similarity between certain commercial batches due to their physicochemical quality; therefore, the inclusion of this parameter in the evaluation allows for a reduction in the number of classes established by the physical classification; (ii) the commercial batches classified as Type 1 and Type 2 presented low reflectance in the NIR and SWIR regions, indicating greater interaction with the chemical composition of the grains and, consequently, a greater presence of compounds associated with nutritional and sensory quality; (iii) the MLP model presented high performance for the classification task, with metrics above 97% and strong consistency between the evaluated folds, but it did not differ statistically from the CAT, LGBM, XGB and RF models, which also achieved metrics above 95–96%; and (iv) the SWIR region demonstrated greater discriminatory relevance, highlighting the contribution in 2173 nm spectral point to the differentiation of batches of polished white rice.

## Figures and Tables

**Table 1:** Machine learning and regression models used for the classification of grains of white polished rice.

Acronym	Models	Reference
RF	Random Forest	[6]
GB	Gradient Boosting	[30]
SVM	Support Vector Machine	[31]
KNN	K-Nearest Neighbors	[3]
MLP	Multilayer Perceptron	[23]
XBG	Xtreme Gradient Boosting	[32]
LGB	Light Gradient Boosting Machine	[33]
CAT	Categorical Boosting	[34]
LR	Logistic Regressor	[24]

**Table 2:** Hyperparameters used for each machine learning model in the rice classification task.

Model	Hyperparameters
CAT	depth = 6; learning_rate = 0.05; n_estimators = 800; random_seed = 42
GB	n_estimators = 200; learning_rate = 0.1; max_depth = 4; random_state = 42
KNN	n_neighbors = 7; weights = “distance”
LGBM	n_estimators = 800; learning_rate = 0.05; num_leaves = 31; subsample = 0.8; colsample_bytree = 0.8; random_state = 42
LR	solver = “lbfgs”; multi_class = “multinomial”; class_weight = “balanced”; max_iter = 5000; random_state = 42
MLP	hidden_layer_sizes = (128, 64); activation = “relu”; solver = “adam”; max_iter = 5000; random_state = 42
RF	n_estimators = 200; class_weight = “balanced”; random_state = 42; n_jobs = −1
SVM	kernel = “rbf”; C = 10; probability = True; class_weight = “balanced”; random_state = 42
XGB	n_estimators = 500; max_depth = 6; learning_rate = 0.05; subsample = 0.8; colsample_bytree = 0.8; reg_lambda = 1.0; random_state = 42

**Table 3:** *p*-values, means, normality and homocedasticity of variables of the centesimal composition of phisycal defects and sound grains in white polished rice.

Sample	Moisture	Starch	Protein	Lipids	Fiber	Ash
	(%)	(%)	(%)	(%)	(%)	(%)
Healthy grains	12.71 a	66.64 a	9.46 e	2.21 c	1.24 h	1.34 d
Broken	12.73 a	66.65 a	8.77 g	2.17 d	1.29 g	1.38 d
Burnt	12.57 b	60.95 e	10.29 b	2.12 d	2.10 a	1.81 a
Pitted or spotted	12.74 a	63.67 c	9.99 c	2.25 c	1.89 b	1.58 b
Streaked	12.53 b	65.38 b	9.08 f	2.13 d	1.61 d	1.51 c
Green	12.01 d	61.32 d	12.57 a	2.74 b	1.71 c	1.79 a
Yellow	12.59 b	63.66 b	9.91 c	2.82 a	1.35 f	1.56 b
Chalky	12.27 c	65.56 b	9.71 d	2.22 c	1.47 e	1.54 b
Pr>Fc	0.0000 *	0.0000 *	0.0000 *	0.0000 *	0.0000 *	0.0000 *
CV (%)	0.26	0.28	0.54	1.35	1.51	1.38
SD (%)	0.24	2.12	1.11	0.27	0.29	0.16
Shapiro–Wilk (*p*)	0.226	0.145	0.641	0.055	0.536	0.883
Levene (*p*)	0.948	0.873	0.641	0.922	0.678	0.727
Average	12.52	64.23	9.97	2.33	1.58	1.56

* significant at *p* < 0.05; CV: coefficient of variation; SD: standard deviation. Means followed by the same letter belong to the same statistical group and therefore do not differ significantly according to the Scott–Knott test at the 5% probability level.

**Table 4:** *p*-values, means, normality and homocedasticity of variables of the centesimal composition of samples from Type 1 to Type 5 and Off-Type white polished rice.

Sample	Moisture	Starch	Protein	Lipids	Fiber	Ash
	(%)	(%)	(%)	(%)	(%)	(%)
Type 1	11.13 d	73.39 a	8.05 c	1.55 c	2.10 a	1.12 c
Type 2	11.84 c	71.65 b	8.84 b	1.43 d	2.04 b	1.18 a
Type 3	12.43 b	70.54 c	9.31 a	1.45 d	1.98 c	1.19 a
Type 4	12.50 b	70.09 d	9.47 a	1.45 d	1.92 d	1.18 a
Type 5	13.06 a	69.24 e	9.57 a	1.61 b	1.86 e	1.16 b
Off-Type	12.40 b	70.92 c	8.63 b	1.71 a	1.99 c	1.18 a
Pr>Fc	0.0000 *	0.0000 *	0.0000 *	0.0000 *	0.0000 *	0.0000 *
CV (%)	6.81	1.95	9.81	13.70	5.88	4.26
SD (%)	1.03	1.90	1.02	0.23	0.14	0.05
Shapiro–Wilk (*p*)	0.787	0.758	0.827	0.545	0.040	0.000
Levene (*p*)	0.951	0.695	0.729	0.863	0.701	0.136
Average	12.23	70.97	8.98	1.53	1.98	1.17

* significant at *p* < 0.05; CV: coefficient of variation; SD: standard deviation. Means followed by the same letter belong to the same statistical group and therefore do not differ significantly according to the Scott–Knott test at the 5% probability level.

## Data Availability

The original contributions presented in this study are included in the article. Further inquiries can be directed to the corresponding author.
